# Comparative evaluation of antifungal susceptibility testing methods for *Rhizopus* species isolates

**DOI:** 10.22034/cmm.2024.345165.1480

**Published:** 2023-12

**Authors:** Mohd Saqib Hasan, Prashant Gupta, Gopa Banerjee

**Affiliations:** Department of Microbiology, King George’s Medical University, Lucknow, India

**Keywords:** Antifungal susceptibility testing, Disc diffusion, E test, *Mucorales*, *Rhizopus*

## Abstract

**Background and Purpose:**

The mainstay of treatment for COVID-19-associated mucormycosis was liposomal Amphotericin B. Other antifungal agents, such as posaconazole and isavuconazole, were used as well. The Clinical and Laboratory Standards Institute (CLSI) and the European Committee on Antimicrobial Susceptibility Testing recommend broth microdilution methods for antifungal susceptibility testing. In this regard, the present study aimed to see what potency and zone diameters correlate with the gold standard broth microdilution method.

**Materials and Methods:**

All the isolates were identified by matrix-assisted laser desorption ionization–time-of-flight. In total, 127 isolates of 83 *Rhizopus oryzae* complex and 44 isolates of *Rhizopus microsporus* complex were selected. Anti-fungal susceptibility testing by disc diffusion and E-test was performed on Mueller Hinton Agar and compared with the CLSI broth microdilution method of Anti-fungal susceptibility testing.

**Results:**

Percentage agreement was found to be more in the case of the E test than the disc diffusion method. In the case of *R. oryzae*, posaconazole had 98.79% agreement with broth microdilution followed by Isavuconazole (97.59%), Itraconazole (96.38%), and Amphotericin B (91.56%).

**Conclusion:**

Disc diffusion correlates well with broth microdilution, although its correlation is weaker when compared to the E test. Effective concentration of Amphotericin B discs for antifungal susceptibility testing depends on the specific *Rhizopus* species.

## Introduction

Mucormycosis, an infrequent, yet life-threatening fungal infection caused by various *Mucorales* species, primarily affects individuals with compromised immune systems [ 1 ]. Patients infected with *Mucorales* fungi face a mortality risk of up to 50% [ 2 ]. As of February 2021, India experienced a dramatic surge in daily COVID-19 cases, marking the onset of the second wave of the pandemic. The causes behind this resurgence were multifaceted [ 3 ]. Moreover, the geographical distribution of this wave varied significantly among states, with some being hit much harder than others [ 4 ].

In the context of COVID-associated mucormycosis, the primary treatment has been liposomal Amphotericin B (L-AMB) [ 5 ]. The L-AMB stands out as the preferred choice due to its lower nephrotoxicity, superior tissue penetration, and higher tissue concentration [ 6 , 7 ]. Other antifungal agents, such as posaconazole (PSC) and isavuconazole (ISC), have also been employed, either as the first line of drug when L-AMB was not an option or as salvage and step-down therapy.

Antifungal susceptibility testing (AFST) is crucial in guiding the choice of appropriate treatment. While standards, such as the Clinical and Laboratory Standards Institute (CLSI) and European Committee on Antimicrobial Susceptibility Testing (EUCAST), recommend the broth microdilution method (BMD) for AFST, it is predominantly conducted in referral laboratories or advanced facilities. In contrast, AFST using the disc diffusion (DD) method offers a simpler and more accessible option for most clinical microbiology labs. However, CLSI does not provide specific guidelines regarding optimal disc potencies or zone diameter breakpoints for Amphotericin B (AMB), PSC, ISC, and Itraconazole (ITZ) against *Mucorales*.

With these considerations in mind, this study aimed to determine the potency and zone diameter values that correlate with the gold standard BMD.

## Materials and Methods

This study was carried out in the postgraduate Department of Microbiology, King George Medical University, Lucknow, India for 18 months from May 2021 to October 2022. This study was approved (Ref. code: PGTSC-IIA/P29) by the Ethics Committee of King George Medical University.

In total, 127 out of 203 culture-positive *Mucorales* were confirmed as *Rhizopus* isolates (83 *Rhizopus oryzae* and 44 *Rhizopus microsporus*) by matrix-assisted laser desorption ionization–time-of-flight (MALDI-TOF, Vitek MS, Biomeriux, France). They were tested using lactophenol cotton blue for AMB, PSC, ISC, and ITZ by CLSI M38 A3. Simultaneously, they were tested by E test (Biomeriux, France) for AMB and PSC; however, the E test strips were not available for ISC and ITZ. Furthermore, they were tested by DD (as per CLSI M51A) for all the drugs with two different concentrations. Discs were prepared in-house with the following concentrations: AMB 10 µg (low concentration disc [L]) and 20 µg (high concentration disc [H]), PSC and ISC 5 (L) and 10 µg (H), and ITZ 10 (L) and 30 µg (H). The incubation time was 24 h according to the optimal incubation time suggested by Espinel-Ingroff et al. [ 8 ].

Minimum inhibitory concentration (MIC) value interpretation of BMD for wild type (WT) and non-wild type (non-WT) was based on the epidemiological cut-off values (ECV) suggested by Espinel-Ingroff et al. and P. Gupta et al. [ 9 , 10 ]. Therefore, derived from available data, the reference cut-off values of *R. oryzae* and *R. microsporus* for AMB were 4 and 2 µg/mL, respectively. Similarly, the cut-off values of *R. oryzae* and *R. microsporus* for PSC were 2 µg/mL. Finally, the cut-off values of *R. oryzae* and *R. microsporus* for ITZ were 2 and 8 µg/mL, respectively. For ISC, it was reported at 4 µg/mL for both species. Zone diameter breakpoints were determined for WT depending on the zone diameters ≥ 95% of the isolates for all drugs. Since MIC breakpoints are not available, the ECV suggested by Espinel-Ingroff et al. [ 9 ] and Gupta et al. was considered for analysis [ 10 ].

## Results and Discussion

The disc diffusion method is an easier and more reliable alternative to broth microdilution for routine diagnostic laboratories. Although fungal infections are on the rise in India, data on antifungal susceptibility, especially for *Mucorales* are lacking. The present study showed that the DD method of Antifungal susceptibility may also be used to test *Mucorales*. The DD method has already been validated for other filamentous fungi, but the breakpoints for *Mucorales* have never been suggested in CLSI M 51A, CLSI M61, and EUCAST 2022.

Based on the findings of the present study, if a low-concentration disk of AMB is used in *R. oryzae*, the zone diameter cut-off should be 14 mm. This zone diameter corresponds to MIC of 4 μg/ml. The *R. microsporus* cut-off value is similar, which is 14 mm, but it corresponds to MIC of 2 μg/ml. For low concentrations of ITZ, the zone diameters for *R. oryzae* and *R. microsporus* are suggested to be 20 and 13 mm, respectively. The ITZ zone diameters of both species correspond to MIC of 4 μg/ml. For low concentrations of PSC, the zone diameter of 13 mm is suggested which corresponds to MIC of 2 μg/ml for both species. In the case of low-concentration ISC, 24 mm zone diameter is suggested for *R. oryzae* and 23 mm for *R. microsporus*. It should be mentioned that both of these zone diameters correspond to MIC of 4 μg/ml (Table 1). 

**Table 1:** Percentage of agreement of disc diffusion for wild-type and non-wild-type strains of *Rhizopus* species isolates

Wild-type strains of *Rhizopus*
Antifungals	*Rhizopus* species	Numbers of isolates	DD disparity with BMD		Percentage of agreement	
			L*	H*	L*	H*
Amphotericin B	*Rhizopus oryzae*	83	7	6	91.5	92.2
	*Rhizopus microsporus*	42	6	4	85.7	90.4
Itraconazole	*Rhizopus oryzae*	82	2	4	97.5	95.1
	*Rhizopus microsporus*	34	0	0	100	100
Posaconazole	*Rhizopus oryzae*	83	1	3	98.7	96.3
	*Rhizopus microsporus*	44	7	6	84.0	86.3
Isavuconazole	*Rhizopus oryzae*	82	1	2	98.7	97.5
	*Rhizopus microsporus*	44	3	2	95.3	95.4
**Non-Wild-type strains of *Rhizopus***						
Antifungals	*Rhizopus species*	Numbers of isolates	Disc diffusion disparity with BMD		Percentage of agreement	
			L*	H*	L	H
Amphotericin B	*Rhizopus microsporus*	2	0	0	100	100
Itraconazole	*Rhizopus microsporus*	10	2	2	80	80
Itraconazole	*Rhizopus oryzae complex*	1	1	1	0	0
Isavuconazole	*Rhizopus oryzae complex*	1	1	1	0	0

DD: disc diffusion, BMD: broth microdilution method

In other studies, Salas et al. and Espinel-Ingroff et al. have suggested 15 mm as the zone diameter cut-off for AMB in the case of *Mucorales* which contrasts with the findings of the present study [ 8 , 11 ]. This difference may be due to a smaller number of isolates and geographically different isolates used by them. In the present study, the disparity of zone diameter with BMD for ITZ was very low in the WT strains of both *R. oryzae* and *R. microsporus*. This contrasts with the findings of a study carried out by Espinel-Ingroff et al. which showed the lowest reproducibility of *Rhizopus* spp. with ITZ (Table 1). 

Few studies have considered a zone diameter cut-off of 13 mm for PSC as resistant [ 11 ]. However, in the present research, it was found that the majority of isolates of *Rhizopus* spp. clustered at this zone diameter. This zone diameter also corresponded with a MIC value of 2 µg/mL. This deviation could be due to the different geographical locations of isolates and a single large epidemic of post-COVID mucormycosis in the State of Uttar Pradesh, India.

In this study, only 2 non-WT strains of *R. microsporus* were found for AMB while 10 were found for ITZ. In the case of *R. oryzae*, only 2 non-WT strains were found for ITZ and 1 for ISC (Table 1). None of the strains with MIC above the proposed ECV were found for PSC which contrasts with the results of a study performed by Anuradha et al, who found 26% (6/23) of *R. microspores* PSC MICs above the proposed ECV [ 12 ]. The reason for this difference could be the use of a low ECV MIC value of 1 μg/ml on the smaller sample size by them.

In the present study, the disparity of zone diameter with BMD for ITZ was very low in the case of WT strains of both *R. oryzae* and *R. microsporus*. This is in contrast with the findings of a study conducted by Espinel-Ingroff et al. which showed the lowest reproducibility of *R. species* with ITZ [ 8 ]. A zone diameter cut-off of 13 mm for PSC has been considered resistant by few studies. However, in this study, it was found that the majority of the isolates of *Rhizopus* spp. clustered at this zone diameter. This zone diameter also corresponded with MIC of 2 µg/mL. The difference between the results of the present and previous studies may be due to the different geographical conditions and isolation from a single large epidemic of post-COVID mucormycosis in the present research.

In this study, the percentage agreement ranges for PSC in DD and PSC in E test were 84.09-98.79% and 98.79-100%, respectively (Table 2). A study conducted by Espinel-Ingroff et al. in 2007 also showed an excellent agreement of PSC DD with BMD for filamentous fungi (96-98%) [ 8 ].

**Table 2:** Percentage of agreement of E-test and disk diffusion method (CLSI M51-A) with reference broth micro-dilution (CLSI M38-A2)

Isolates	Amphotericin B		Itraconazole	Posaconazole		Isavuconazole
	E-test	DD	DD	E-test	DD	DD
*Rhizopus oryzae* complex (n=83)	98.7	91.56	96.38	98.7	98.79	97.59
		(L)	(L)		(L)	(L)
		92.27	93.97		96.38	96.38
		(H)	(H)		(H)	(H)
*Rhizopus microsporus* complex (n=44)	97.7	86.36	95.45	100	84.09	93.18
			(L)		(H)	
		90.90	95.45		86.36	95.45
			(H)		(L)	(H)

DD: Disc Diffusion, L: Low concentration potency disc, H: High concentration potency disc

In the present study, a moderate and significant level of correlation was found in all the antifungals with BMD for Mucorales, except a low level of correlation that was found in both concentrations of AMB for *R. oryzae*, both concentrations of PSC for *R. microsporus*, and high concentrations of ITZ for *R. oryzae*.

All the high disc concentrations did not change the result interpretation; therefore, any concentration of the drug can be used. However, lower concentrations of drugs are suggested since these concentrations are used in routine for *Candida* and *Aspergillus*.

There was a positive correlation of antifungal susceptibility of both species by MIC with the E test for AMB and PSC. Level of correlation was found to be moderate for both drugs in the case of *R. microsporus* (‘r’=-0.692-0.825) and *R. oryzae*, mild for AMB (‘r’=0.458), and poor for PSC (‘r’=0.072). Therefore, if available, an E-test may be a better choice in comparison with DD.

## Conclusion

Disc diffusion correlates well with BMD, although its correlation is weaker, compared to the E test. Effective concentration of AMB discs for AFST depends on the specific *Rhizopus* species.

Lower concentrations of discs exhibit better agreement with BMD for PSC, ISC, and ITZ. Disc diffusion is considered an acceptable alternative to BMD. Consequently, DD proves to be a reliable method for AFST of *Rhizopus* isolates in the laboratory. Given the possibility of encountering non-WT strains, every clinical microbiology laboratory must conduct AFST for *Rhizopus* isolates.
